# TYROSINEMIA TYPE III: A CASE REPORT OF SIBLINGS AND LITERATURE REVIEW

**DOI:** 10.1590/1984-0462/2020/38/2018158

**Published:** 2020-06-05

**Authors:** Fábio Barroso, Joana Correia, Anabela Bandeira, Carla Carmona, Laura Vilarinho, Manuela Almeida, Júlio César Rocha, Esmeralda Martins

**Affiliations:** a Centro Hospitalar Universitário do Porto, Porto, Portugal.; b National Institute of Health, Porto, Portugal.

**Keywords:** Tyrosine, Tyrosinemias, Attention deficit disorder with hyperactivity, Metabolism, Tirosina, Tirosinemias, Transtorno do déficit de atenção com hiperatividade, Metabolismo

## Abstract

**Objective:**

Tyrosinemia type III (HT III) is the rarest form of tyrosinemia, and the full clinical spectrum of this disorder is still unknown. The neurological involvement varies, including intellectual impairment and attention deficit disorder with hyperactivity (ADHD). We report the case of two siblings diagnosed with HT III at different ages.

**Case description:**

The index case was diagnosed by newborn screening for endocrine and metabolic disorders, starting a low-protein diet immediately, with a consistent decrease in tyrosine levels. By the age of three, the child displayed a hyperactive behavior, starting treatment for ADHD two years later. At seven years of age, he shows a slight improvement in terms of behavior and attention span and has a cognitive performance slightly lower than his peers, despite maintaining acceptable tyrosine levels. His sister, who had a history of ADHD since age five, was diagnosed with HT III after family screening at the age of eight. Despite initiating a dietetic treatment, her behavior did not improve, and she has a mild intellectual impairment.

**Comments:**

This is the first case report describing siblings with HT III who underwent nutritional treatment with a low-protein diet in different phases of life, with a better neurological and behavioral evaluation in the patient who started treatment earlier.

## RESUMO

**Objetivo:**

A tirosinemia tipo III (TT III) é a forma mais rara das tirosinemias e o espectro clínico desta entidade não está totalmente esclarecido. O envolvimento neurológico é variável, incluindo o atraso cognitivo ou transtorno do déficit de atenção com hiperatividade (TDAH). Descrevemos o caso de dois irmãos que foram diagnosticados com TT III em idades diferentes.

**Descrição dos casos:**

O caso índice foi diagnosticado no contexto do rastreio endócrino-metabólico neonatal, tendo iniciado imediatamente dieta hipoproteica, com redução consistente dos níveis de tirosina. Por volta dos três anos, foi detectado um comportamento hiperativo, tendo iniciado dois anos depois tratamento para o TDAH. Aos sete anos, apresenta leve melhora de comportamento e da atenção e avaliação cognitiva levemente inferior ou pouco abaixo quando comparado a crianças da mesma faixa etária, apesar de manter níveis aceitáveis de tirosina. A sua irmã, com história de TDAH desde os cinco anos, foi diagnosticada de TT III aos oito anos no contexto do rastreio de familiares. Apesar de iniciar tratamento dietético, nenhum efeito foi notado em termos de comportamento e a doente apresenta leve atraso cognitivo.

**Comentários:**

Este é o primeiro caso clínico descrito de irmãos com TT III que iniciaram terapêutica dietética com dieta hipoproteica em diferentes fases da vida, com melhor avaliação em termos neurológicos e comportamentais no doente que iniciou tratamento mais precocemente.

## INTRODUCTION

Tyrosine is a non-essential amino acid, obtained directly from diet or the hydroxylation of phenylalanine. It is a precursor in the synthesis of catecholamines, thyroxine, and melanin.^1^

Tyrosinemia type III (OMIM 276710) is a rare inborn error of tyrosine metabolism caused by mutations in the gene encoding the enzyme 4-hydroxyphenylpyruvate dioxygenase (HPPD), which catalyzes the conversion of 4-hydroxyphenylpyruvate to homogentisate, the second step in the tyrosine catabolic pathway. It is the rarest form of tyrosinemia, being transmitted in an autosomal recessive form. This metabolic disorder is characterized by elevated levels of serum tyrosine and increased excretion of phenolic metabolites [4-hydroxyphenylpyruvate (4-HPP), 4-hydroxyphenyllactate (4-HPL) and hydrophenylacetate] in the urine. Only a few case reports have been described in the literature, with a wide clinical phenotype spectrum: some patients presented neurodevelopmental symptoms while others were diagnosed by newborn screening, being asymptomatic.^1^ ^,^ ^2^ ^,^ ^3^

We report the case of two siblings with tyrosinemia type III who were diagnosed and started treatment at different ages, presenting their clinical outcomes. A brief literature review is also presented.

### CASE DESCRIPTION

#### Case 1

The male patient is the third child from a Portuguese consanguineous couple (third cousins), with an unremarkable family history. He was born at term by vaginal delivery following an uneventful pregnancy, with an Apgar score of 9 and 10 at 1 and 5 minutes, respectively. His birth weight was 3,510 g (89^th^ percentile), length 50 cm (76^th^ percentile), and head circumference 35.5 cm (93^rd^ percentile).

The newborn screening performed at the fifth day of life revealed elevated tyrosine levels (526 µmol/L; cut-off values: <248 µmol/L). A control sample taken at the 29^th^ day showed the persistence of elevated plasma tyrosine levels (680 µmol/L), with high urinary excretion of 4-HPL and 4-HPP and presence of N-acetyl-tyrosine and vanillactic acid.

The child started a protein-restricted diet supplemented with a tyrosine- and phenylalanine-free amino acid mixture at 1-month-old. Subsequent metabolic controls showed a consistent decrease in tyrosine levels: 280 µmol/L at 1.5 months, 262 µmol/L at 7 months, and 165 µmol/L at 12 months.

The diagnosis of tyrosinemia type III was confirmed by a genetic analysis performed by amplifying exons one to 14 of the HPD gene using polymerase chain reaction followed by DNA sequencing, which demonstrated a homozygous mutation p.A33T (c.97G>A).

His physical examination was normal, particularly with no ocular or skin involvement, and presenting typical growth (weight curve at the 85‒97^th^ percentile until 30 months and then at the 50‒85^th^ percentile; height curve at the 50^th^ percentile until 30 months and then at the 50‒85^th^ percentile; normal head circumference). His neurological examination was normal, and he had a brain magnetic resonance imaging at the age of 30 months showing no abnormalities. His blood test analysis (full blood count, liver and renal function, and electrolytes) was unremarkable.

His early psychomotor development was normal, with head control at less than two months, sitting alone without support at approximately eight months, and walking alone at 12 months. First words were spoken at 12 months, and he constructed simple sentences at the age of 18 months. However, after the age of three, language development progressed slowly, with sound articulation problems.

The parents also reported a hyperactive behavior, with impulsivity and inability to follow orders by the age of three. Throughout his pre-school years, he was repeatedly considered incapable of following the level of learning of his classmates.

A formal developmental assessment using the Griffiths Mental Development Scale at 32 months showed a global developmental quotient (GDQ) of 88.9, and the test was repeated at 54 months showing a GDQ of 87 (slightly lower score compared to the children in his age group, with more evident difficulties on the sub-scale of hearing and language).

At the age of five, the neurodevelopmental unit diagnosed him with Attention Deficit Disorder with Hyperactivity (ADHD) with a combined subtype (DSM-V criteria), and he initiated treatment with methylphenidate. He also started speech therapy sessions.

**Figure 1:**
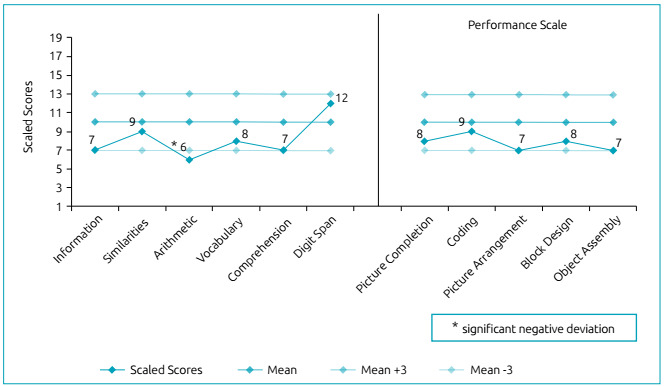
WISC-III subtest profile of patient 1.

Currently, at the age of seven, he shows a slight improvement in terms of behavior and attention span and still undergoes speech therapy. He attends elementary school (2^nd^ grade) with no learning difficulties, and at his last formal developmental assessment (Wechsler Intelligence Scale for Children^®^, third edition, Portuguese version), he had an intelligence quotient (IQ) of 78, a verbal IQ of 81, and a performance IQ of 82 (Figure 1).

His tyrosine levels have been consistently below 300 µmol/L. On his last nutritional status evaluation performed at the age of seven, he presented a daily natural protein intake of 1 g/kg, supplemented with 1.1 g of amino acids/kg from phenylalanine- and tyrosine-free amino acid mixtures. Body composition analysis was considered adequate.

#### Case 2

A 15-year-old female, the eldest sibling of patient 1, was diagnosed with tyrosinemia type III at eight years of age, after a family screening following her brother’s diagnosis.

She was born at term by cesarean section (pelvic presentation) following an uneventful pregnancy, with an Apgar score of 8 and 9 at 1 and 5 minutes, respectively. Her birth weight was 4,030 g (87^th^ percentile), length 53 cm (87^th^ percentile), and head circumference 36 cm (83^rd^ percentile). Her neonatal period was unremarkable, as was her early psychomotor development. She had a history of primary nocturnal enuresis and vesical instability, treated with desmopressin and oxybutynin hydrochloride since the age of six.

She was diagnosed with ADHD at the age of five and treated with methylphenidate and risperidone. She presented learning difficulties when she attended elementary school (2^nd^ grade), and was included in a special education program at the age of ten.

At the time of diagnosis, her initial tyrosine level was 1,769 µmol/L. On the first nutritional status evaluation, her daily natural protein intake was 1.4 g/kg. This value dropped to 1.08 g/kg, and she started taking supplementation with 0.7 g of amino acids/kg from phenylalanine- and tyrosine-free amino acid mixtures, enabling a consistent decrease of tyrosine levels below 300 µmol/L.

Her physical examination and growth were normal (weight and height at the 50‒85^th^ percentile), with an unremarkable neurological examination. The genetic study revealed the same mutation as her brother in homozygosity.

**Figure 2:**
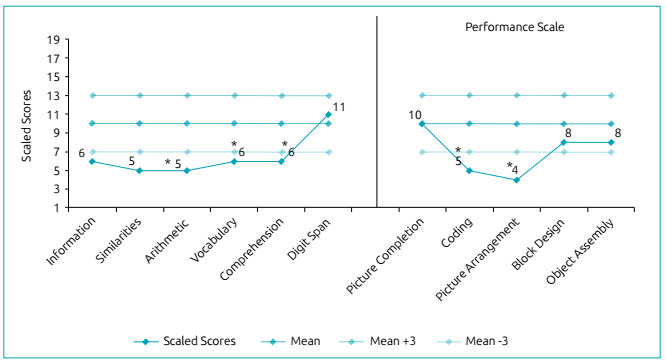
WISC-III subtest profile of patient 2.

After initiating a low-protein diet, her behavior and school performance did not improve. The minimum level of daily natural protein intake reached was 0.65 g/kg at 14 years of age. She is currently in 7^th^ grade, still in the special education program, and under treatment with methylphenidate. Her latest formal developmental assessment (Wechsler Intelligence Scale for Children^®^, third edition, Portuguese version) revealed a global IQ score of 68, with a verbal IQ of 68 and a performance IQ of 77 (Figure 2).

She slowly began diet liberalization and is now with a daily natural protein intake of 0.9 g/kg, combined with 0.8 g of amino acids/kg, maintaining tyrosine levels below 300 µmol/L nevertheless.

## DISCUSSION

The symptoms of tyrosinemia type III are not well characterized, and there is no apparent correlation between tyrosine serum levels, the clinical phenotype, and the mutation type. As in the two cases presented, many patients have neurodevelopmental manifestations, including intellectual impairment, learning difficulties, dyslexia, attention deficit, hyperactivity, behavioral disturbance, ataxia, microcephaly, hypotonia, and seizures, but a classical phenotype has not been described.^1^ ^,^ ^2^ ^,^ ^3^

Contrary to tyrosinemia types I and II, patients do not demonstrate any evidence of hepato-renal dysfunction or skin or eye lesions. In 2015, an 11-year-old girl with normal development and no neurological signs was diagnosed with tyrosinemia type III following the investigation of recurrent proteinuria (9-17 mg/L); in this case, laboratory tests revealed elevated serum tyrosine levels, which led to the diagnosis of the disease. However, it was not clear if the nephrological complications were associated with tyrosinemia type III.^4^

In a 2001 review, which included 13 patients, the most common long-term complication was intellectual impairment (75% patients). Five patients were diagnosed by newborn screening, three of whom started a low-tyrosine and -phenylalanine diet after diagnosis; among them, two had no symptoms and presented normal development at 13 months and five years and five months, respectively, while the third had a delayed psychomotor development but demonstrated an average developmental quotient at the age of four (Griffiths Mental Development Scale). One patient only started treatment at 8 months of age, when developmental retardation was detected, and another who was not treated showed intellectual impairment. Eight patients were diagnosed after the neonatal period, seven of them because of neurologic signs and one due to developmental delay. Only one patient had normal development at 17 years of age.^2^

The etiology of the neurological manifestations is not known but could be related to hypertyrosinemia as in tyrosinemia types I and II. Tyrosine and/or its derivatives seemed to be neurotoxic metabolites, and mental delay was associated with increased plasma concentrations of these substances. Several studies revealed that hypertyrosinemia inhibits the functioning of respiratory chain complexes, compromises the Krebs cycle, and decreases creatine kinase and pyruvate kinase activities, inducing an oxidative stress status and an impairment of energy metabolism in the cerebral cortex of rats.^5^ ^,^ ^6^ ^,^ ^7^ ^,^ ^8^

Patients detected by newborn screening appear to have fewer neurological symptoms and a lower degree of cognitive impairment compared to those diagnosed later in life.^2^ ^,^ ^3^ However, it is unclear if the clinical outcome is determined by the decrease in plasma tyrosine levels, and the neurological evolution can vary despite similar tyrosine levels.^2^ Besides, there are asymptomatic patients diagnosed later in childhood or adolescence who never developed neurological or behavioral symptoms.^4^

Another proposed hypothesis is that neurological impairment could be explained by an excessive nitric oxide release, which could contribute to neuronal damage.^9^

Although it is unclear if lowering tyrosine levels can alter the natural history of the disease, treating it with a low-protein diet, at least in early childhood, to maintain tyrosine levels between 200 and 500 µmol/L has been considered reasonable.^10^

Both our patients started nutritional treatment after diagnosis, maintaining tyrosinemia levels below 300 µmol/L afterward. However, while one was detected by newborn screening, the other was eight years old at the time of diagnosis and already symptomatic with learning difficulties. Clinically, they both presented ADHD requiring pharmacological treatment, but patient 1 had a better cognitive outcome despite being below average compared to the healthy population group. He attends the 2^nd^ grade with a normal curriculum.

In patient 2, despite initiating treatment, her behavior did not improve, and she has a mild intellectual impairment. A decision to gradually increase natural protein intake was made, and she has maintained acceptable tyrosine levels, although she still takes phenylalanine- and tyrosine-free amino acid supplements to satisfy her nitrogen needs. These findings have been reported, and many patients seem to be able to maintain these levels after childhood, without diet control.^2^

Currently, a significant number of metabolic disorders related to major psychiatric diseases remain underdiagnosed for years before more specific organic signs become evident. Metabolic diseases most associated with ADHD are succinic semialdehyde dehydrogenase deficiency, phenylketonuria, X-linked ichthyosis, and mucopolysaccharidosis type III (Sanfilippo syndrome).^11^

Establishing a genotype-phenotype correlation in tyrosinemia is difficult since the literature has very few cases described on the subject, and only some of them identify distinct mutations. Tables 1 and 2 summarize the main clinical and analytical characteristics of patients known to date.

**Table 1:** Summary of patients with tyrosinemia type III detected by neonatal screening.

Case-study	Gender	Age at diagnosis	Family history	Form of presentation	Neurological abnormalities	Brain image	Plasma tyrosinemia at diagnosis	Genetic study	Treatment	Follow-up
Patient 1	Male	Neonatal screening	Consanguineous parents	Asymptomatic	Speech delay, ADHD	Normal	526 µmol/L	Homozygous for A33T mutation in the HPD gene	Low-Phe/Tyr diet	“Borderline” development (7 y)
1- Preece et al.^12^	Female	Neonatal screening	Crouzon syndrome	Asymptomatic	Developmental delay at 8 mo	Not described/performed	355 µmol/L	Not described/performed	Low-Phe/Tyr diet	Learning difficulties (7 y) Crouzon syndrome
2- Standing et al.^13^ and Rüetschi et al.^14^	Female	Neonatal screening	First cousin of patient 3 (Table 1) Consanguineous parents	Asymptomatic	Mild jitteriness, brisk tendon reflexes	Abnormal appearance in subcortical and brainstem white matter (30 mo)	1094 µmol/L	Homozygous for Y258X mutation in the HPD gene	Low-Phe/Tyr diet	Delayed psychomotor development (5 y)
3- Standing et al.^13^ and Rüetschi et al.^14^	Male	Neonatal screening	First cousin of patient 2 (Table 1) Consanguineous parents	Neonatal hepatitis	None	Not described/performed	Range: 500-1000 µmol/L in the first 19 mo	Homozygous for Y258X mutation in the HPD gene	Normal diet	Mild intellectual impairment (17 y)
4- Ellaway et al.^15^	Not described	Neonatal screening	Not relevant	Asymptomatic	None	Not described/performed	937 µmol/L	Not described/performed	Low-Phe/Tyr diet	Normal development (13 mo)
5- Ellaway et al.^15^	Female	Neonatal screening	Not relevant	Asymptomatic	None	Not described/performed	581 µmol/L	Not described/performed	Low-Phe/Tyr diet	Normal development (5 y 5 mo)
6- Heylen et al.^10^	Male	Neonatal screening	Consanguineous parents	Asymptomatic	None	Not described/performed	398 µmol/L	Homozygous splice donor mutation in intron 11, IVS11+1G>A in the HPD gene	Low-Phe/Tyr diet	Normal development (30 mo)

ADHD: attention deficit disorder with hyperactivity; HPD: hydroxyphenylpyruvate dioxygenase; mo: months; Phe: phenylalanine; Tyr: tyrosine; y: years.

**Table 2:** Summary of patients with tyrosinemia type III detected after the neonatal period.

Case-study	Gender	Age at diagnosis	Family history	Form of presentation	Neurological abnormalities	Brain image	Plasma tyrosinemia at diagnosis	Genetic study	Treatment	Follow-up
Patient 2	Female	8 y	Consanguineous parents	Learning difficulties	Psychomotor retardation, ADHD	Not described/performed	1769 µmol/L	Homozygous for A33T mutation in the HPD gene	Low-Phe/Tyr diet	Mild intellectual impairment (15 y)
1- Endo et al.^16^	Male	Infancy	Parents were siblings mother had hypertyrosinemia	Seizures and pneumonia at day 21 of life	Seizures, encephalopathy	Mild cerebral atrophy	640 µmol/L	Not described/performed	Low-Phe/Tyr diet	Died due to accidental asphyxiation (day 105)
2- Giardini et al.^17^ and D’Eufemia et al.^18^ ^,^ ^19^	Female	17 mo	Not relevant	Acute ataxia, confusion, motor incoordination, hypotonia, absent tendon reflexes	Recovered from all symptoms	Not described/performed	624 µmol/L	Not described/performed	Low-protein diet until 3 y	Autoimmune thyroiditis (9 y) Normal development (17 y)
3- Cerrone et al.^3^ and Rüetschi et al.^14^	Male	3.5 y	Not relevant	Developmental delay from 8 mo	Psychomotor retardation, hyperactivity, self-injurious behavior, severe speech delay	Normal	532 µmol/L	Heterozygous for Y200X and I335M mutations in the HPD gene	Low-Phe/Tyr diet until 12 y	Severe intellectual impairment (14 y)
4- Tomoeda et al.^20^	Male	7 weeks	Not relevant	Neonatal restlessness, reduced head control	Microcephaly	Not described/performed	594 µmol/L during treatment	Homozygous for A268V mutation in the HPD gene	Low Phe/Tyr diet until 17 y, then low-protein diet	Moderate intellectual impairment (19 y)
5- Rüetschi et al.^14^	Male	7.5 y	Brother of patient 4 (Table 2) Consanguineous parents	Urolithiasis	Essential tremor	Not described/performed	830 µmol/L	Homozygous for Y160C mutation in the HPD gene	Normal diet	Mild intellectual impairment (7.5 y) Lost to follow-up
6- Rüetschi et al.^14^	Male	18 y	Brother of patient 5 (Table 2) Consanguineous parents	Psychomotor retardation	Essential tremor	Not described/performed	262 µmol/L	Homozygous for Y160C mutation in the HPD gene	Normal diet	Lost to follow-up
7- Ellaway et al.^15^	Male	14 y	Consanguineous parents	Mild psychomotor retardation, generalized seizures	Suspected seizures since 2 y	Not described/performed	913 µmol/L	Not described/performed	Low-protein diet initially, then ceased	Intellectual impairment (18 y)
8- Ellaway et al.^15^	Not described	18 mo	Not relevant	Developmental delay	None	Not described/performed	1305 µmol/L	Not described/performed	Low-Phe/Tyr diet	Mild global developmental delay (6 y)
9- Tahiroglu et al.^21^	Male	2 y	Not described	Developmental delay from 15 mo	Psychomotor retardation, hyperactivity, autistic symptoms	Not described/performed	“Elevated levels”	Not described/performed	Low-Tyr diet	Severe intellectual impairment (9 y) Persistence of hyperactivity
10- Szymanska et al.^4^	Female	11 y	Not described	Recurrent proteinuria since 7 y	None	Not described/performed	439 µmol/L	Homozygous for T160C mutation in the HPD gene	Normal diet	Normal development (11 y)

ADHD: attention deficit disorder with hyperactivity; HPD: hydroxyphenylpyruvate dioxygenase; mo: months; Phe: phenylalanine; Tyr: tyrosine; y: years.

To our knowledge, this is the first case report of siblings with tyrosinemia type III who underwent nutritional treatment with a low-protein diet in different life stages, with the one who started it earlier and during an asymptomatic phase showing better results in terms of neurological and behavioral outcomes.

Both patients presented ADHD as a neurological manifestation. Therefore, we emphasize the importance of conducting a metabolic study in children with this disorder who do not respond adequately to pharmacological treatment.

Although the pathophysiology of neuronal injury in tyrosinemia type III is not completely explained by the accumulation of tyrosine in the central nervous system, a restrictive tyrosine and phenylalanine diet is recommended during childhood. Further studies and collection of information on these patients are necessary to understand the consequences of HPPD deficiency, the mechanisms of brain injury, and the long-term outcome in patients with this rare form of tyrosinemia.
